# How Classy Servant Leader at Workplace? Linking Servant Leadership and Task Performance During the COVID-19 Crisis: A Moderation and Mediation Approach

**DOI:** 10.3389/fpsyg.2022.810227

**Published:** 2022-03-23

**Authors:** Muhammad Zada, Shagufta Zada, Mudassar Ali, Zhang Yong Jun, Nicolás Contreras-Barraza, Dante Castillo

**Affiliations:** ^1^Business School, Henan University, Kaifeng, China; ^2^Department of Management Sciences, Alhamd Islamic University, Islamabad, Pakistan; ^3^Department of Business Administration, Faculty of Management Sciences, Ilma University, Karachi, Pakistan; ^4^Department of Management Science, Capital University of Science and Technology, Islamabad, Pakistan; ^5^Facultad de Economía y Negocios, Universidad Andres Bello, Viña del Mar, Chile; ^6^Centro de Estudios e Investigación Enzo Faletto, Universidad de Santiago de Chile, Santiago, Chile

**Keywords:** crisis, COVID-19, servant leadership, task performance, psychological empowerment, virtual work environment, perceived supervisor support

## Abstract

The COVID-19 pandemic has caused a record global crisis, particularly and extremely, for the service sectors. Due to extensive security measures, many service sector employees have to work remotely to maintain services. Drawing upon the conservation of resources theory, this research investigates the impact of servant leadership on the task performance of employees in virtual working environments during the COVID-19 crisis. Our theoretical model was tested using data collected from 335 individual employees in the education sector of Pakistan. SPSS version 26.0 was applied to find the hypothesized relationship between the study variables. To find the indirect mediating effect, we applied Model 4; for moderation, we applied Model 1; and for the moderation and mediation effect, we applied Model 7 of the Process Macro model of Hayes. The results of the study revealed that servant leadership is positively related to task performance in a virtual environment during crises. Furthermore, psychological empowerment partially mediates the relationship between servant leadership and task performance. Perceived supervisor support positively moderates the relationship between servant leadership and task performance. Moreover, the indirect effect of servant leadership on task performance *via* psychological empowerment is moderated by perceived supervisor support. The results provided guidance to the educational sector on how to lead effectively in times of crisis when service sector employees work predominantly in virtual environments. The theoretical and practical implications of these findings are discussed.

## Introduction

COVID-19 affects all sectors of a country that directly contribute to the country’s economy and development, and it pushes countries into a global crisis. One of the most affected sectors is the education sector which is an important sector of the country (Haroon and Rizvi, 2020). Educational institutions were closed for a long period of time during the worldwide pandemic (Craven et al., 2020). Similar effects were observed in Pakistan where schools, colleges, and universities were closed as per the government’s orders causing severe hurdles for the education ministry to operate universities and colleges in usual ways (Finsterwalder and Kuppelwieser, 2020; Kabadayi et al., 2020). As a result, they shifted mainly to the online system and worked from home to keep things going. Unfortunately, the sudden shift from physical to online was hard to incorporate for the teachers and students. They were facing a significant level of technical issues and found it challenging to adapt to the new learning system. Many students reported financial crises during the pandemic, preventing them from paying school fees. It was reported that the family income sources from services such as airlines, hotels, and hairdressers possibly could not offer their services because of lockdown restrictions. Families that depended on these income resources lost their earning opportunities; as a result, they were unable to financially support their dependents.

Contrarily, the educational sector continued operating by setting a virtual work environment to sustain its operations (Demir et al., 2020). An effective work environment refers to employment settings where workers are designated at different levels geographically, technically, and mentally to keep the organizational mission and goals (Huang et al., 2010). Throughout the pandemic leaders faced problems with making organizational changes and sustaining their quality performance (Collings et al., 2021). Leaders were not prepared for this sudden change and considered it as an imposed change that had negatively affected the task performance of employees operating in the educational sector (Aboramadan et al., 2020; Carnevale and Hatak, 2020). Considering the decline in performance, the present study will investigate the relationship between leadership style and the degree of decisive performance in such situations. In this respect, a conceptual model will be developed to investigate the extent to which servant leadership affects the task performance of employees operating in a virtual work environment in the educational sector. This study will find the mediating role of psychological empowerment and also the moderating role of supervisor support. In this context, it was hypothesized that servant leaders will enhance and sustain the task performance of employees to result in high productivity and high performance in the virtual work environment.

The study aims to contribute to the past literature of management by scrutinizing four major issues. First, the challenges confronting managers about the virtual work environment in the school setting have not been adequately addressed, offering little empirical evidence regarding it. Keeping in view these facts, the current study emphasizes and examines the influence of the servant leadership style on employee performance in a virtual environment which employees are not used to, but must adapt to during the emergency of COVID-19 within the Pakistani context. Previous literature found that important characteristics such as employee performance (Liden et al., 2014), team effectiveness (Hu and Liden, 2011), employee creativity (Aboramadan, 2020), and positive workplace behavior (Brière et al., 2021) are influenced by servant leadership. Yet, very few studies have focused on the psychological empowerment perspective. On the basis of previous literature, the present research proposes the underlying mechanism of employee psychological empowerment which refers to four cognitive states: meaning, self-assurance, competence, and those that reflect on an individual’s orientation to their work (Spreitzer, 1995). The components of psychological empowerment enhance the competency of subordinates which are beneficial for identifying the entire efforts to accomplish the defined goals (Chiang and Hsieh, 2012). Past research has explicitly highlighted a significant relationship between leadership and psychological empowerment in typical circumstances (Jeung and Yoon, 2016; Chen et al., 2018).

Second, the power-sharing behavior of leaders is significantly related to the motivation and performance of employees for the accomplishment of collective goals that eventually enhance workplace performance (Chua et al., 2012; Khan et al., 2022). Employees who feel that their work and efforts are being admired are motivated to perform better (Liden et al., 2000; Ali et al., 2020). Literature supports the notion that servant leadership can psychologically empower the employees by enhancing their strength, commitment, and contribution to the organizational goals and recognize their ideas to accomplish and execute tasks in virtual work settings. Subsequently, psychological empowerment potentially intervenes and links servant leadership with the task performance of employees during the COVID-19 crisis. This is uniquely fascinating as the unexpected change to a virtual workplace due to the COVID-19 pandemic creates family, work, and life problems and other difficulties, whether operating at an individual or a group level (Kabadayi et al., 2020).

Third, empirical studies addressing the association between leadership and employee job performance during a crisis are limited (Uhl-Bien and Arena, 2018). The present research probes the moderating effect of supervisor support on how servant leadership could improve the task performance of employees. Moreover, prior literature found that the COVID-19 pandemic seems to be the primary reason behind the rapid digital transformation (Bartsch et al., 2020). In this vein, it is expected that the organization’s expertise will significantly influence the effectiveness of the services of the servant leadership in the education sector.

Fourth, the main objective of the present research is to empirically scrutinize the extent to which the servant leadership style influences the task performance of employees in the virtual workplace. However, this is the first study to contribute to literature on managing education and leadership in virtual work environments during the COVID-19 crisis. This article relates the professional insights learned on how to influence employee task performance and production that mostly concerns the leaders in the work environment, especially in a period of crisis. The two theories, the theory of conservation for resources (COR) and social exchange theory (SET), are used as the theoretical basis for the research of this article. Under the study of the direct outcome of servant leadership on the work performance of employees, it is used to more clarify the research. Furthermore, the indirect influence of servant leadership on the task performance of an employee within the virtual workplace during COVID-19 is not studied in prior literature. The literature is also missing the underlying mechanism such as psychological empowerment and how perceived supervisor support may positively influence and enhance the task performance of employees of the education sector in Pakistan.

### The Literature and Conceptual Background

The literature about the challenges of the education sector and the COVID-19 crisis has emphasized work environments, work climate, and the newness of the issue for the target population and researchers (Alsabbagh and Khalil, 2016; Herhausen et al., 2017; Alavi et al., 2018). Liao (2017) supports that the practices, challenges, and behaviors of workgroups operating in the virtual work environment to date have been inadequately explored. This unique business climate is pressuring businesses to rebuild their business model and procedures to acquire and sustain their competitive edge in the modern cooperate world, which is highly virtual. To grapple with this rapidly changing environment, the leadership plays a vital role in building employee task performance. However, very few studies have been conducted on the knowledge of the precursor of the virtual work environment leadership style to deal with this rapidly evolving work climate. The leadership seems to play a considerable part in improving the task performance of subordinates. In any case, not many studies have explored the forerunner of leadership style in the virtual workplace. In this context, scarcely any empirical work has been found in the education sector in a virtual setting. Few studies address this subject matter, analyzing, for instance, challenges faced by the student population (Gedro et al., 2020), job satisfaction (Mihhailova et al., 2011; Madlock, 2018), precursors and outcomes of team effectiveness (Schepers et al., 2011; Zhang et al., 2015), and power, management, and workplace politics (Muganda and Pillay, 2013) in the digital corporate world. There is a gap in literature on the impact of leadership style on overall subordinate performance at a time of a high level of uncertainty in the education sector. Stoker et al. (2019) seem to be exempt as the study has reflected upon the leadership style and behavior in the manufacturing and financial sectors during the 2008 economic crisis. Results revealed that directive leadership significantly increased employee task performance, whereas no effect was found in the participative leadership practices in the targeted sectors. This reflects the significance of undertaking task-oriented behaviors and practices in emergency circumstances, yet their level of significance is not known to date.

In conclusion, we found that there have been no studies addressing the role of servant leadership in the education sector at the time of crisis during the COVID-19 pandemic. However, to fill the literature gap, the primary function of a recent study is the call to examine leadership practices that reduce fear among the employees regarding extreme work outcomes (Hannah et al., 2009; Tecco, 2020). This current study invested efforts to investigate the outcome of servant leadership (SL) styles on worker performance during the virtual workplace in the crisis due to COVID-19. Stoker et al. (2019) differentiated between the directive and participative leadership in detail. The emphasis of the current study will remain on the “task dichotomy and relation-oriented leadership behaviors” to investigate two divergent forms of management behavior. We built the current study model, which gave a helpful hypothetical framework to recognize main supervisors’/leaders’ states, procedures, mediators and moderators, and results pertinent to task performance in the virtual work settings (Dulebohn and Hoch, 2017).

### Hypothesis Model

**FIGURE 1:**
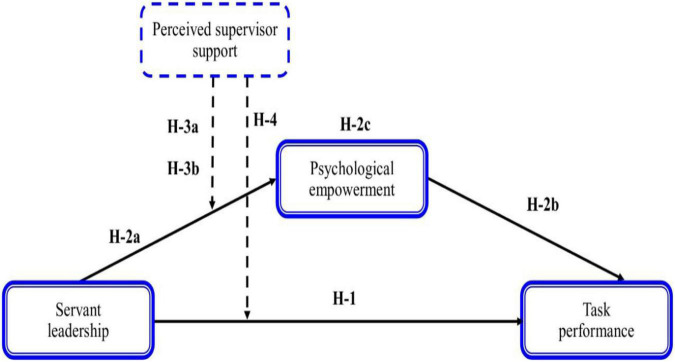
Research model and hypotheses.

Servant leadership practices improve individual and group outcomes in virtual workplaces (Liao, 2017). Leaders find it difficult to inspire, explain, and motivate geologically scattered employees (Berson et al., 2015). Hence, to empower serving styles and adaptability, influential in a virtual setting of work, they are supposed to emphasize the development and enhancement of the self-administration capacities of their subordinates (Carte et al., 2006). Further, in a virtual workplace, organization leaders struggle to effectively coordinate and communicate with their associates because of differences in working hours or other additional burdens, since it is apparent for the duration of the crisis by COVID-19. Employees may need guidance in comprehending and completing the assigned tasks (Liao, 2017). In this manner, leaders ought to take a step in dealing with the team projects virtually since they are familiar with the objectives, assets, and processes to accomplish the team task (Liao, 2017). The various difficulties related to the virtual working setting, especially during a pandemic crisis, require supervisors and leaders to adopt a servant leadership style for sustaining team performance (for example organizing tasks and empowering employees). Subsequently our model recommends that servant leadership prompts task performance through psychological empowerment. The moderating role of perceived supervisor support is helpful in the relationship of servant leadership with task performance and psychological empowerment (Figure 1).

### Impact of Servant Leadership on Task Performance

There is a significant role for a leader to contribute to accomplishing employees’ daily tasks within an organization (Lee et al., 2019; Aboramadan and Dahleez, 2021). The behavior of how leaders deal with the situations they face defines their attributes of leadership (Nifadkar et al., 2012). Leadership refers to influencing others to get the work done effectively and efficiently (Andriani et al., 2018). According to Ridgeway (2003), leadership means the leader’s ability to encourage others to achieve organizational goals. Many researchers observe that the performance of the employees is influenced by the leadership directly and indirectly (MacKenzie et al., 2001). Leadership is a complicated process that involves combining and integrating individual commitments, inputs, and accomplishments into a helpful collective endeavor (Spreitzer and Cameron, 2012; Ali et al., 2018). Among the numerous leadership styles, servant leadership has drawn more recently significant attention among both academics (Van Dierendonck, 2011; Liden et al., 2014) and practitioners (Spears, 1995) in response to a growing interest in a more ethical, pro-social, and people centered management leadership style. Servant leadership is described as an endeavor that develops and strengthens employees through a leader’s benevolence, sympathy, feeling of morals, and stewardship (Greenleaf, 2002; Liden et al., 2008). Servant leadership is unique in that servant leaders identify their followers’ needs, empower them, and provide opportunities to achieve performance (Van Dierendonck, 2011). The core objective of servant leadership is to appreciate the individuals, encourage them, value their efforts, perform task performance, provide directions to the followers, help them, and counsel them. According to Van Dierendonck and Patterson (2015), servant leadership focuses on a strong bonding between the leaders and followers. Servant leaders focus on psychological factors of care, safety, security, confidence, and equality (Yoshida et al., 2014) in a work context (Peoples, 2021). Research has shown that servant leaders represent a high degree of people orientation. It is believed that the freedom of work will help employees to maximize their ability to work that would lead to a mutual benefit of both the organization and the employee.

Moreover, leaders who adopt a servant leadership attitude are focused on workers’ activities and provide joint support which enhances their emotional state and increases task performance of the employees (Liden et al., 2015). Therefore, threats related to innovative work behavior would be reduced, and valuable support is provided to employees to enhance their task performance (Hu and Liden, 2011; Yoshida et al., 2014; Aboramadan and Dahleez, 2021). Thus, servant leadership is encouraged for connecting with employees and enhancing task performance among workers.

**H1.** There is a positive relationship between servant leadership and employee task performance in virtual teams during the COVID-19 crisis.

### The Mediating Role of Psychological Empowerment

Psychological empowerment is a combination of four dimensions that reflect a person’s direction toward his or her work that includes meaning, affect, competence, and self-assurance. This meaning refers to the extent to which an employee aligns their job role, values, beliefs, own convictions, qualities, and norms (May et al., 2004). Competence is an individual’s perception of self-adequacy about fruitful achievement (Bandura, 1986). Self-assurance indicates the independence enjoyed by an individual to pick a job for themselves (Thomas and Velthouse, 1990; Zhang, 2015). Affect reflects the extent to which employees feel that their work and effort endeavor to impact and accomplish the task at hand (Spreitzer, 1995).

Servant leaders can empower their employees psychologically by prompting different aspects of self-concepts (Owens et al., 2013; Sousa and van Dierendonck, 2017). For instance, servant leadership recognizes and accepts the commitment of the workers to the executive aim and objectives (De Clercq et al., 2014; Mallén et al., 2019), and treats workers with respect and empathy, which gives them a ground perceive their efforts and work as regarded and effective for organizational success (Chen et al., 2018; Wang and Yin, 2020). Moreover, servant leaders pay attention to their subordinates’ opinions and ideas that upgrade their certainty and self-confidence about their job positions, improve their competency, and further develop team performance (Hunter et al., 2013). Furthermore, the assignment of power and receptiveness to criticism shown by fair leaders liberate the employees’ experiences by starting practical limitations and allowing them to independently operate where they can create a difference in the accomplishment of work targets (Owens and Hekman, 2012).

Psychologically empowered employees are characterized by devotion and flexibility with expanded endeavors toward task accomplishment, inspiration and motivation for their job role (Seibert et al., 2011; Sun et al., 2020). Psychologically empowered workers accept extra roles and duties and become more autonomous, which are the primary markers of managerial sustainability and customer contentment (Namasivayam et al., 2014; He et al., 2021). In an organization’s development process, staff can operate autonomously along with external authoritative commands of leadership. There is intensifying proof that independent, permanent, and trusted employees can achieve the targets in a given time frame (Scott-Young and Samson, 2008). Employees who have self-confidence are quite sure about their success and performance (Zhang and Bartol, 2010). Subordinates knowing about the influence of their work task endorse the feeling that their task job is sufficient (McColl-Kennedy and Anderson, 2002; Khattak et al., 2018) which turns into a motivational instrument for them to apply to other endeavors toward project achievement (Aga et al., 2016). The literature revealed that empowered workers are extra proficient in achieving tasks and facilitating to accomplish managerial aims and objectives (Sigler and Pearson, 2000; Khattak et al., 2020) and enhance task performance (Laschinger et al., 2009). Similarly, psychologically empowered employees can primarily add to the accomplishment of undertaking team/group tasks with great performance, thus adding to the goal accomplishment allocated to the team.

The previous studies propose that psychological empowerment intervenes between servant leadership style and employees task performance. Furthermore, our argument for the mediating effect of psychological empowerment is based on the concept of conservation of resources theory (Hobfoll, 2011b). To adopt the conservation of resources theory, this study addresses that the servant leadership role is to serve others and provide maximum benefits to the employees (Hobfoll, 2011a). It is expected that servant leaders spend their assets to create more resources for their subordinates which empower them and incline them to perform well. Therefore, psychological empowerment appears to influence the impact of servant leadership for task performance.

Research indicates that psychological empowerment has three factors that include: competence, meaning, and self-determination which lead to task performance (Sigall and Mills, 1998). The previous studies strongly argue that psychological empowerment significantly impacts the shared variance of servant leadership and worker task presentation. On the basis of the aforementioned literature, we propose the following hypothesis:

**H2a.** Servant leadership is significantly positively related to psychological empowerment in virtual teams during the COVID-19 crisis.
**H2b.** Psychological empowerment is significantly positively related to team performance in virtual teams during the COVID-19 crisis.
**H2c.** Psychological empowerment mediates the relationship between servant leadership and team performance in virtual teams during the COVID-19 crisis.

### Perceived Supervisor Support as a Moderator

Chen and Wu (2020) indicated that perceived supervisor support is the employees’ perception that provides a favorable serving environment. Thus, perceived supervisor support plays an essential role in giving organizational resources and incentives to subordinates and should be viewed as a significant source of support. Such support pertains to how employees see their supervisor appreciating their work and efforts and showing concern for their well-being (Eisenberger et al., 2002). It is considered a significant amplifier of establishing a supportive workplace during crises (Rhoades and Eisenberger, 2002; Farid et al., 2021). In the face of high vulnerability, employees might encounter job insecurity. In such situations, they tend to seek their manager’s valuable help in evaluating and adapting to the uncertain crisis (Lee and Peccei, 2007; Tian et al., 2014). For this situation, supervisors might give tangible support to their subordinates and offer psychological help to reduce their mental pressure (Kossek et al., 2018). In particular, supervisors might assist their subordinates in reframing their insecurities regarding their job perspectives so that it feels less unpleasant and urges them to embrace positive and versatile conduct and behavior at the workplace (Cooke et al., 2019; Khan et al., 2021). Subsequently, it is assumed that a leader’s support adds to the positive feeling of employees and enhances task performance (Cole et al., 2006; Rathi and Lee, 2017) and positively affects emotional commitment (Saeed et al., 2015; Li et al., 2018). Employees will be more emotionally dedicated to their respective organizations when they see their supervisor supporting them in positive directions (He et al., 2020; Ali et al., 2021). Besides, the adverse consequence of job uncertainty on emotional commitment is more remarkable when the work environment supports a lower level of supervisory support than when there is a more elevated level of it. Supervisor support reduces the adverse consequences of job insecurity on the employees’ emotional states and increases the number of employees.

We apply this notion to examine the relationship between servant leaders and the task performance of employees. Servant leadership reflects the leader’s productive attitude toward task accomplishment and performance. On the other hand, supervisor support inspires employees to act reasonably toward their supervisor/leader by reciprocating positively. Furthermore, psychological empowerment significantly affects the employee’s task performance (Feiz et al., 2019). Moreover, it positively affects the performance of the project units, and hence the individual interests of its supervisors. Consequently, supervisor support comprises a working climate that energizes employees through psychological empowerment. Furthermore, workers who see themselves with strong supervisor support in the organization will tend to develop positive psychological empowerment (Rhoades and Eisenberger, 2002). Employees who feel strong support from the organization side will be more psychologically empowered and this will enhance their task performance. Interestingly, when workers observe supervisor support at lower levels, task performance and efforts might decrease in task accomplishment.

In more broad terms, examination of the supervisor support indicates that it has a direct influence on employee task performance. For example, McGovern et al. (1997, p. 12) stated that the line manager’s behavior and practice may deform, and possibly even challenge the commitment for achieving the managerial goals. Similarly, Coyle-Shapiro and Shore (2007) revealed that the line manager’s job might be complying with or breaking the conditions of the more distal trade with the respective firm. Moreover, line supervisors have figured out how to invest time or interest in executing tasks (Purcell et al., 2008). On account of leadership, subordinates might encounter low degrees of help for participating in such activities although they are relied upon to do so. Therefore, they may experience mental stress and anxiety (Park and Jang, 2017), which could block or mitigate task performance. Also, research demonstrates that manager support impacts the connection between task performance, cooperation, and influence in the work settings. However, lower levels of perceived supervisor support might endorse the feeling that employees are less creative and show low-level of growth. Conclusively, employees perceive a lower level of support from their immediate line managers due to an absence of help from the association. Employees who have a given lower level of perceived supervisor support will show smaller amounts of commitment, which decreases psychological empowerment and task performance (Rhoades and Eisenberger, 2002).

Thus, employees who notice lower levels of supervisor support may believe that they are not fairly compensated since duties are perceived to be imposed on them and may react by lowering their work performance. On the basis of prior literature, it is hypothesized that:

**H3a.** Perceived supervisor support (PSS) moderates the association between SL and task performance such as if there is a high level of PSS there will be a high level of task performance and vice versa in virtual teams during the COVID-19 crisis.
**H3b**. Perceived supervisor support moderates the association between servant leadership and “psychological empowerment,” i.e., a higher perceived supervisor support strengthens the relationship in virtual teams during the COVID-19 crisis.

**H4**. An indirect effect of servant leadership on task performance through mediating role of psychological empowerment is moderated by perceived supervisor support, such as high PSS lead to high task performance in virtual teams during the COVID-19 crisis.

## Methodology

### Research Context and Sample

To investigate these hypotheses, we gathered online data from the service sector of Pakistan that primarily offers education services (e.g., in schools, colleges, and universities). The data collection proceeded during the middle of the lockdown, December 2020 to August 2021, when the education system service quickly changed to virtual workplaces. All these aspects make it more appropriate for the current study. Initially, 450 questionnaires were distributed among the employees of the service sector. Out of 450 questionnaires, 365 were received with 81.1% response rate. Out of 365 questioners, 30 questionnaires were missing values and were not appropriate to include in this study. So, we entertained only 335 questionnaires with a response rate of 74.44% as a final sample for this study. The demographic results show that 32 respondents were from 20 to 30 years of age (9.5%), 95 respondents were 31–40 years of age (28.4%), 111 respondents were from 41 to 50 years of age (33.1%), and 97 participants were above age 51 (29%). Further, demographic results show that 225 participants (67.0%) were master’s degree holders and 110 respondents (32.7%) were Ph.D. degree holders. Furthermore, demographic results show that 33 respondents (9.85%) have 1–5 years of experience, 52 respondents (15.52) have 6–10 years of experience, 78 respondents (23.29%) have 11–15 years of experience, and 172 respondents (51.34%) have above 16 years of experience. Moreover, the demographic result revealed that 289 respondents (86.26) were from public sector universities, and 46 respondents (13.73) were from private sector universities.

### Measure

#### Servant Leadership

To estimate the influence of servant leadership, a scale given by Liden et al. (2015) was employed in the current study. It consists of seven items from good to excellent. Cornbach alpha value was α = 0.95. The items included scrutinized the leadership effectiveness from the employee’s perspective, such as “My supervisor makes my career development a priority.”

#### Task Performance

To measure the effectiveness of the job performance a 7-item scale given by Williams and Anderson (1991) was used with a reliability index of α = 0.79. A 5-point Likert Scale was utilized to verify the response starting from “strongly disagree (1) to strongly agree (5).” The items mainly address the task performance, “This employee adequately completes assigned duties” and “Meets formal performance requirements of the job.”

#### Psychological Empowerment Scale

Spreitzer (1995) developed a comprehensive short scale for measuring psychological empowerment based upon a 12-item scale. The items were similar to “You are confident about your ability to do your job.” The alpha reliability value of the items was α = 0.93. Moreover, a 5-point Likert scale was used i.e., 1 for strongly disagree and 5 for strongly agree.

#### Perceived Supervisor Support

Supervisor support was evaluated through a short scale based on four items of the “Spanish version of the Supervisor Support scale” developed by Kottke and Sharafinski (1988). The instrument mainly emphasizes the employees perceived support from their immediate supervisors. This support could be tangible and intangible. The items included are: “My supervisor is concerned about the welfare of those under him/her,” “My supervisor pays attention to what I’m saying,” “My supervisor is successful in getting people to work together” and “My supervisor helps get the job done.” The scale uses a-point Likert scale varying from 1 = strongly disagree to 4 = strongly agree” with a reliability index of α = 0.90.

#### Control Variable

To enhance the validity of the results, confounding variables were controlled that includes gender, age, education level, and job experience. These variables were found to potentially affect the task performance (Bartsch et al., 2020).

## Results

### Common Method Bias

In the crosssectional study, a common method bias mostly occurs. Harman’s single factor is among the used tests to check for common method bias (Podsakoff, 2003). The Harman’s single factor shows 31.8% variation extracted (Cut-Off value = 50%). Non-response bias is calculated by taking the first and last values of the variables of the data but the relation was insignificant, so a non-response bias does not exist (Table 1).

**TABLE 1:** Factor loadings.

Constructs	Items	Factor loadings	Cronbach’s alpha	CR	AVE
	SL1	0.87			
	SL2	0.65			
	SL3	0.77			
Servant leadership	SL4	0.77	0.84	0.86	0.50
	SL5	0.52			
	SL6	0.72			
	SL7	0.62			
	PSS1	0.77			
Perceived supervisory support	PSS2	0.78	0.70	0.84	0.57
	PSS3	0.75			
	PSS4	0.73			
	PE1	0.76			
	PE2	0.76			
	PE3	0.79			
	PE4	0.75			
	PE5	0.84			
Psychological empowerment	PE6	0.87	0.70	0.95	0.64
	PE7	0.91			
	PE8	0.70			
	PE9	0.87			
	PE10	0.74			
	PE11	0.68			
	PE12	0.79			
	TP1	0.92			
	TP2	0.84			
	TP3	0.76	0.78	0.93	0.67
Task performance	TP4	0.87			
	TP5	0.67			
	TP6	0.92			
	TP7	0.74			

Table 2 shows that servant leadership is significantly correlated with perceived supervisor support (*r* = 0.12, *p* < 0.001), psychological empowerment (*r* = 0.16, *p* < 0.001), and task performance (*r* = 0.17, *p* < 0.001). Perceived supervisor support is also significantly correlated with psychological empowerment (*r* = 0.43, *p* < 0.001), task performance (*r* = 0.69, *p* < 0.001), and psychological empowerment is also significantly correlated with employee task performance (*r* = 0.54, *p* < 0.001). Furthermore, the reliability of the main variables of the study shows that all variables have a good reliability value, i.e., all variables have a reliability greater or equal to 0.70. Furthermore, the control variables show no effect.

**TABLE 2:** Mean, standard deviation, and correlation results.

Var	Mean	SD	1	2	3	4	5	6	7	8	9
(1) Gender	1.46	0.49	1								
(2) Age	2.05	0.92	0.010	1							
(3) Education	2.22	0.82	–0.054	–0.017	1						
(4) Experience	2.58	0.89	–0.028	0.024	–0.017	1					
(5) Sector	0.51	0.50	–0.032	–0.086	–0.057	–0.012	1				
(6) SL	3.24	1.16	0.041	–0.065	–0.001	–0.040	–0.011	0.84			
(7) PSS	3.09	1.03	0.053	–0.085	–0.018	–0.035	–0.010	0.853**	0.70		
(8) PE	3.49	1.06	0.077	−0.123*	0.008	–0.043	0.024	0.770**	0.781**	0.70	
(9) TP	3.15	1.03	–0.019	–0.013	0.100	−0.108*	–0.075	0.690**	0.571**	0.571**	0.78

**p < 0.01, *p < 0.05, SL, servant leadership, PSS, perceived supervisory support, PE, psychological engagement, TP, task performance.

### Mediation Analysis

Psychological empowerment regulated the mediating role from servant leadership to task performance. As shown in Table 3, psychological empowerment partially mediates the relationship between servant leadership and task performance (BootLLCI = 0.1452 and BootULCI = 0.3158). As zero is not contained in the 95% confidence interval for the indirect effect, this supports the study’s fourth hypothesis (H2c). Our mediation model explained approximately 56% of the variance between servant leadership and task performance.

**TABLE 3:** Mediation effect.

	Boot LLCI	Boot ULCI	Boot SE	β	Decision
Mediation path	0.1452	0.3158	0.0444	0.2245	Partial mediation

**FIGURE 2:**
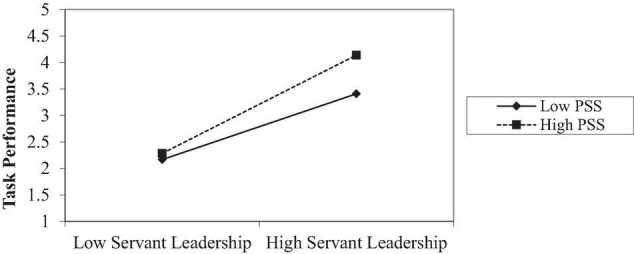
SL × PSS = Task performance.

**FIGURE 3:**
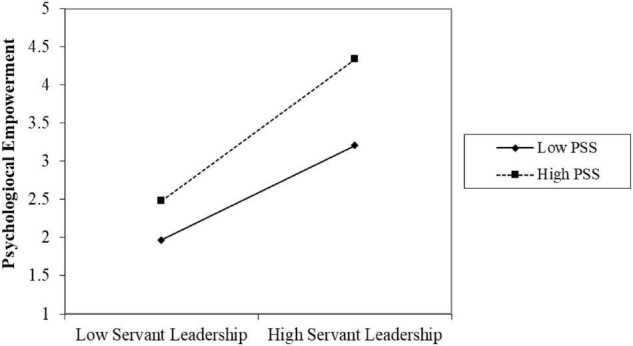
SL × PSS = Psychological empowerment.

Hayes (2017) Process Macro Model 1 has been applied to test the moderation. Table 4 shows the moderating effects of perceived supervisory support on the relationships between servant leadership and task performance (*b* = −0.0594, SE = 0.0258, *t* = −2.3049, *p* = 0.0218, [LLCI = −0.1101 ULCI = −0.0087], which supports the H3a Hypothesis (see Figure 2). Table 5 shows the moderating effect of perceived supervisory support on the relationship between servant leadership and psychological empowerment (*b* = −0.1532, SE = 0.0356, *t* = −4.3070, *p* = 0.0000, [LLCI = −0.2231 ULCI = −0.0832], supporting the H3b Hypothesis (see Figure 3).

**TABLE 4:** Moderation effect on task performance.

Model	B	se	T	*p*	LLCI	ULCI
Constant	0.1501	0.3688	0.4069	0.6843	–0.5755	0.8756
Servant leadership	0.3509	0.1058	3.3176	0.0010	0.1429	0.5590
P. supervisor support	0.8240	0.0933	8.8332	0.0000	0.6404	1.0075
Interaction	–0.0594	0.0258	–2.3049	0.0218	–0.1101	–0.0087

**TABLE 5:** Moderation effect on psychological empowerment.

Model	B	se	T	*p*	LLCI	ULCI
Constant	–0.7923	0.5090	–1.5567	0.1205	–1.7935	0.2089
Servant leadership	0.7748	0.1460	5.3080	0.0000	0.4877	1.0620
P. supervisor support	1.0118	0.1287	7.8600	0.0000	0.7585	1.2650
Interaction	–0.1532	0.0356	–4.3070	0.0000	–0.2231	–0.0832

### Moderation Mediation Analysis

Moderated-mediation analysis reveals the process in which the mediating variable, psychological empowerment, depends on the value of a moderating variable i.e., perceived supervisory support. To check the moderated mediation, we evaluated the integrated model whereby the strength of the relationship between servant leadership and task performance through psychological empowerment is conditional on the value of a moderator i.e., perceived psychological empowerment. The moderated mediation is proved when the conditional indirect effect of servant leadership on task performance in the presence of perceived supervisory support is significant. The interaction effect of servant leadership and perceived supervisory support on task performance through psychological empowerment is significant as the conditional indirect effect is significant (BootLLCI = −0.0706 BootULCI = −0.0150, *p* < 0.01) as revealed by bootstrapped results (see Table 6).

**TABLE 6:** Bootstrapped results of moderated mediation.

	Index	BootSE	BootLLCI	BootULCI
P. supervisor support	−0.0404	0.0141	−0.0706	−0.0150

## Discussion

Although researchers investigated the relationship between leadership and accomplishment of the planned organizational tasks, results revealed that change appears at multiple levels, for example Battilana et al. (2010); Oreg and Berson (2019); Sverdlik et al. (2020). However, there have been no studies to investigate the role of servant leadership in achieving desired employee performance and organizational results within a high level of uncertainty and crisis as is the case of COVID-19, specifically in a Pakistani context. Consequently, this study has made significant theoretical contributions by reflecting upon the significance of the servant leadership style in enhancing employee task performance in the virtual work climate, keeping in view the challenges posed to the service sector during the pandemic crisis. This research also emphasized the mediating responsibility of psychological empowerment connecting servant leadership and task performance. The moderation analysis was also carried out to investigate that perceived supervisory support moderates the relation between the independent and dependent variables of the study. The data were gathered from the employee population operating in the service sector of Pakistan. The outcomes uncovered that direct and indirect servant leadership styles (through psychological empowerment) strengthen job performance. The results of this study filled a significant breach in the literature *via* reflecting upon the association concerning servant leadership and task performance of employees operating in the virtual workplace during the COVID-19 pandemic. The findings of the recent study propose that serving behavior ought to be a fundamental aspect for the line managers to guarantee fruitful execution of the task, supporting the past studies addressing the topic under scrutiny of ordinary circumstances (Liden et al., 2014). In addition, given the effect of servant leadership in task accomplishment and performance, this study supports past research corroborating an affirmative relation connecting servant leadership and task performance (Saleem et al., 2020). Moreover, the research shows an underlying mechanism to investigate the function of leadership styles such as servant leadership and task performance (Turner and Müller, 2005).

Expanding on the study model (Liao, 2017), we reflected upon the moderating and mediating factors during a broad pandemic in a virtual workplace. Current research suggests that psychological empowerment works as a mediating mechanism between servant leadership and task performance. Moreover, the direct effect of servant leadership on task performance can be increased when employees become psychologically empowered in a virtual work setting. This finding is in line with the results of the past studies that servant leadership positively affects employee task performance through psychological empowerment (Chen et al., 2018). This result likewise reveals that when servant leaders direct their associates, they believe themselves to be mentally engaged and authorized because they benefit from the independence in their job beneath the management of servant leaders (Jeung and Yoon, 2016). Along these lines, employees feel committed and cheered to achieve the organizational goals (Binyamin and Brender-Ilan, 2018). In general, the results of the current study propose that the psychological empowerment of the workers is a valuable instrument that could potentially enhance the task performance of employees that could be accomplished by team leaders who are well-equipped with serving traits.

This study acknowledged the moderating effect of perceived supervisor support on the association of servant leadership and the task performance of employees. Results support the past literature indicating that supervisor support significantly moderates the relationship between servant leadership and task performance (Kanwal et al., 2017). This conclusion additionally shows that servant leaders and support from the supervisor help achieve high task performances. The findings show that perceived supervisor support significantly moderates the association between servant leadership and psychological empowerment.

Furthermore, the study investigated if the perceived supervisor support moderated the relationship between servant leadership and task performance and psychological empowerment (Boonstra, 2013). It has been found that supervisor support could be utilized to establish and sustain a servant leadership style where employees feel their feedback is esteemed and appreciated. Sustainable supervisory support builds up the inspiration to take part in and exert efforts in accomplishing shared objectives and common goals. When employees receive feedback from their supervisor or from a group, they might be ready to participate and show a better performance.

### Practical Implications

Although research examining the role of servant leadership in the context of normal planned organizational change exists (Parris and Peachey, 2013; Harju et al., 2018; Brière et al., 2021), studies on leadership, educational intuition, and service literature dealing with effective servant leadership behavior in a rapid and unpredictable organizational transformation, such as the COVID-19 pandemic crisis, are very scarce. Therefore, our research has certain theoretical and practical implications that contribute to the scarcity of literature on the effectiveness of leadership in educational institutions during a crisis in virtual work environments. First, this paper reveals that servant leadership signifies various resources that are crucial in supporting service employees with task fulfillment while working virtually in times of crisis. Theoretically, the paper is among the few studies to examine the significance of servant leadership in a time of crisis. Furthermore, drawing on the conservation of resource theory (COR), this study addresses a specific mechanism (psychosocial empowerment and perceived supervisor support) through which servant leadership influences task performance.

We examined servant leadership in intuitive educational settings during the pandemic, which is considerably more complex than in normal times due to the sad change in the work environment. The results have revealed that this crisis could cause severe disruptions in the service sector. Eventually, the servant leadership style is unequivocal in sustaining employees’ task performance as it offers them firm grounds to deal with uncertain situations. In crisis times, servant leadership creates harmony among employees and as a result, gets better performances. We have shown that servant leader positive serving behavior contributes to employee’s task performance. Supervisors driving teams through an emergency or crisis should consider this by actively engaging in a serving conduct that gives direction and sets a clear path to be followed by the subordinates that could potentially lead to better performance outcomes in a quickly arising virtual workplace. Encouraging their subordinates, the vital independence and support that empower them to adjust in critical situations or crises turn out best for every individual to accomplish tasks productively.

### Managerial Implications

First, organizations should encourage servant leadership, which can assist workplaces in dealing with the current crisis in a virtual environment. Second, organizational leadership should recognize the productive role of PSS. This key mechanism replicates a positive relationship between organizational leadership and their employee task performance not only for employees in normal working environments but also for those working remotely in virtual working environments. Servant leaders should convey that the organization acknowledges employee contributions, cares about employee well-being, and translates their rhetoric into specific policies and practices. Third, educational institutions both public and private sectors should offer management training programs with courses that emphasize on servant leadership, while policymakers should promote (e.g., flexible work schedules, digital literacy courses, and childcare provisions). To improve task performance and achieve organization goals within the Pakistani context, a country particularly hard hit by a decade of financial crisis before the current COVID-19 pandemic, the above suggestions should be put into practice. We suggest that nurturing servant leadership and PSS are instrumental in bettering such indicators and limiting some of the problems observed in the labor market, such as undeclared work and unpaid overtime (Kumar Bandyopadhyay et al., 2020).

### Limitations and Future Research Directions

The present study has some limitations, despite having several strengths. First, this research used crosssectional primary data and could not draw cause-and-effect conclusions. Future research, therefore, may consider longitudinal studies. Second, we employed a quantitative research methodology. Future research is encouraged to apply qualitative methods to clarify the causality of the relationships between the research variables. Third, while some measures were employed to reduce the common method bias, our study used data collected from one source at one point in time. Considering this, future studies are invited to collect time-lagged data from employees at different points. Fourth, this research investigated the relationships between servant leadership and task performance, psychological empowerment and servant leadership, and perceived supervisor support in the crisis. Future research may consider other outcome variables such as knowledge-sharing behaviors, helping behaviors, and innovative work behaviors. Fifth, in the current study, we investigated the impact of servant leadership style on the outcome variables therefore, future research may consider other leadership styles such as humble, inclusive, ethical transformational, spiritual, and transactional leadership to investigate their comparative effectiveness to servant leadership in crises. Lastly, the study sample was solely collected from Pakistan. This indicates that the study results could not be generalized to the foreign population. Pakistani society is more collectivistic and thus seems familiar with the dependency on the interpersonal relationships prevailing within an organization. In this vein, the significance of servant leadership and perceived supervisor support is more striking in Pakistani culture. Future researchers could conduct a crosscultural study exploring the effect of culture on the leadership processes, employee voice, and their performance. Eventually, our study results show that a servant leader can play an active role in the psychological empowerment of the employees during a crisis. Studies in the future might study the effect of servant leadership on different knowledge areas of management across psychological empowerment dimensions (goal-setting, role clarification, interpersonal process, and problem-solving). Another fruitful area for future research is examining the impact of servant leadership on task performance during the crisis through various mediating/moderating variables such as managerial skills and team building.

## Conclusion

Overall, the results of this study present important theoretical and practical contributions by further exploring SL and other aspects related to employee team performance in a virtual workplace in the context of a crisis such as COVID-19. In particular, we found that SL has a direct and indirect (psychological empowerment) effect on the task performance of employees operating in a virtual work setting during the pandemic crisis. At last, we uncovered that workers having high PSS strengthens the relationship between SL and task performance. We added to literature, knowledge, and understanding about SL and task performance during the crisis.

## Data Availability Statement

The datasets presented in this article are not readily available because due to privacy reasons. Requests to access the datasets should be directed to the corresponding authors.

## Author Contributions

MZ, SZ, and ZJ contributed to the conception and design of the study. MA and MZ organized the database. MA and SZ performed the statistical analysis. MZ, SZ, MA, and ZJ wrote the first draft of the manuscript. DC, MZ, MA, MZ, NC-B, and SZ wrote the sections of the manuscript. All authors contributed to the manuscript revision, read, and approved the submitted version.

## Conflict of Interest

The authors declare that the research was conducted in the absence of any commercial or financial relationships that could be construed as a potential conflict of interest.

## Publisher’s Note

All claims expressed in this article are solely those of the authors and do not necessarily represent those of their affiliated organizations, or those of the publisher, the editors and the reviewers. Any product that may be evaluated in this article, or claim that may be made by its manufacturer, is not guaranteed or endorsed by the publisher.
